# Exploring the diversity and determinants of various depression symptoms in youth: analysis based on the living environments of university students

**DOI:** 10.3389/fpsyt.2025.1672561

**Published:** 2026-01-14

**Authors:** Jinting Wu, Yumei Zhou, Xing Hu, Hairong Liu

**Affiliations:** 1 Clinical Psychology Department, the Second Affiliated Hospital of Wannan Medical College, Wuhu, China; 2 Hospital Sense Department, the Second Affiliated Hospital of Wannan Medical College, Wuhu, China; 3 Dermatology Department, the Second Affiliated Hospital of Wannan Medical College, Wuhu, China; 4 Department of Management, School of Humanities and Management, Wannan Medical College, Wuhu, China

**Keywords:** college students, depression rate, depressive mood, risk factors, sub-threshold depression detection rate

## Abstract

**Objective:**

This study aimed to investigate the prevalence and determinants of depression and subthreshold depression among Chinese university students, with a focus on the influence of demographic, behavioral, and academic factors.

**Methods:**

A cross-sectional survey was conducted among 3,600 undergraduates from five universities in central China using the CES-D scale and a self-designed lifestyle questionnaire. Multivariate logistic regression and ANOVA were employed to identify risk and protective factors.

**Results:**

The overall depression detection rate was 25.60%, with 9.97% classified as subthreshold depression. Male gender, senior year, low family income, and major dissatisfaction were significant risk factors. Regular exercise served as a protective factor, while excessive smartphone use, smoking, alcohol use, and family history of mental illness were associated with increased risk. A dose-response relationship was observed between major satisfaction and depression severity.

**Conclusion:**

The findings support a spectrum-based view of depression and highlight the need for multidimensional, personalized mental health interventions targeting high-risk student subgroups.

## Introduction

Depression is a long-term mood disorder characterized by symptoms such as loss of interest, feelings of guilt, difficulty concentrating, and thoughts of self-harm (1). As a serious health issue, depression not only threatens individual well-being but also significantly increases the global disease burden, affecting health-related life expectancy (2). Research indicates that the high prevalence of depression is one of the major global public health concerns and has broad socio-economic impacts.

University students, being at a critical stage of physical and mental development, typically face challenges such as adapting to new environments, academic pressures, social interactions, and career stress. Additionally, their self-regulation and self-control abilities are not yet fully developed, making them more vulnerable to negative emotions like depression, which may progress into clinical depression. Existing studies show that the incidence of depressive symptoms is relatively high among university students, who are considered a high-risk group for depression (3–5).

Managing depression is a significant challenge, especially given its high relapse rate and the risk of self-harm or suicide. Timely prevention and early identification are crucial to reducing the burden of depression. In recent years, an increasing number of studies advocate for a multidimensional approach to understanding depression, particularly by distinguishing among individuals at different levels of depressive symptoms (6–9). Subclinical depression (SD), the focus of this study, is operationally defined as a condition wherein individuals exhibit notable depressive symptomatology that does not meet the full diagnostic criteria for major depressive disorder. It can manifest as either the presence of all symptoms at a sub-clinical severity level or a subset of clinically significant symptoms (10). Although individuals with subclinical depression exhibit some depressive symptoms, these symptoms are not severe enough to interfere with normal functioning. However, studies suggest that if subclinical depression is not intervened in a timely manner, it may develop into severe depression. The assessment of SD often relies on scale cut-offs, which vary across studies due to the lack of a standardized definition. For instance, while the present study utilized a CES-D score range of 16–19 to define SD, Yin et al. employed a different range (24 to just under 29) (11), reflecting the contextual and methodological influences on SD conceptualization. This variability underscores the need for research that clearly specifies its criteria within the broader spectrum perspective of depression (12). Despite existing research mainly focusing on clinically diagnosed individuals with depression, comprehensive spectrum analysis of various levels of depressive symptoms among university students is relatively lacking (13, 14).

Therefore, this study aims to:(1)Investigate the prevalence of depression and subthreshold depression among Chinese university students using the Center for Epidemiologic Studies Depression Scale (CES-D).

(2)Identify and compare the determinants (including demographic characteristics, academic stress, and health-related behaviors) associated with no depression, subthreshold depression, and clinical depression.

(3)Propose data-driven intervention strategies tailored to the specific risk profiles identified, with a focus on modifiable factors such as physical activity and digital device use.

## Methodology

### Participants and sampling

A cross-sectional survey was conducted from October to December 2022. This study employed a cluster sampling method: first, five universities were randomly selected from central China (including provinces such as Anhui, Hubei, and Henan), and then several classes were randomly chosen from each university. All undergraduate students in the selected classes constituted the target population. A total of 3,600 questionnaires were distributed via the Wenjuanxing platform. After applying specific exclusion criteria (e.g., incomplete questionnaires, incorrect age entries, or inconsistent data), 3,418 valid responses were retained, resulting in a high response rate of 95%. The final sample consisted of 1,353 males (39.58%) and 2,065 females (60.42%). In terms of academic major, the sample comprised1,567 (45.85%)students in medical programs and 1,851 (54.15%) in non-medical programs.

The participants’ ages ranged from 16 to 23 years, with a mean (± standard deviation) of 19.04 ± 1.40 years. In terms of academic seniority (year in university), the sample comprised 1,461 (42.74%) freshmen, 1,214 (35.52%) sophomores, 626 (18.31%) juniors, and 117 (3.42%) seniors.

### Instruments

#### The center for epidemiologic studies depression scale

Depressive symptoms were assessed using the Chinese version of the CES-D scale revised by Zhang Jie et al. This self-report instrument comprises 20 items that measure the frequency of depressive symptoms over the past week on a 4-point Likert scale. The total score ranges from 0 to 60, with higher scores indicating more severe depressive symptoms. Based on established criteria, participants were categorized as follows: a score of ≥20 indicated clinical depression, scores between 16 and 19 denoted subthreshold depression, and scores of ≤15 were classified as non-depressive. In the present study, the CES-D demonstrated good internal consistency, with a Cronbach’s α coefficient of 0.83.

#### Self-designed college student living conditions questionnaire

Utilizing an established survey system and relevant findings, we developed a questionnaire to explore the living conditions of college students. The questionnaire includes general details and everyday behaviors, aiming to gain insights into participants’ personal development, academic pursuits, daily habits, and family backgrounds. The questionnaires are presented in **Table 1**.

**Table 1:** List of variable assignments.

Variable		Assignment
1.	Different depressive symptoms	0= no depression, 1= subthreshold depression, and 2= depression
2.	Sex	0= male, and 1= female
3.	Profession	0= medical class, 1= non-medical
4.	Origin of the student	0= rural, 1= town
5.	Seniority	0= freshman, 1= sophomore, 2= junior, 3= senior
6.	Profession satisfaction	0= satisfied, 1= general, and 2= dissatisfied
7.	Insomnia (Difficulties in falling asleep and staying asleep)	0= None, 1= occasionally (Insomnia is less than 3 nights/month), 3= Often (For 2 nights/week, over 2 months)
8.	Regular Sports activity	0= Yes, 1= No (Physical exercise is 3/week, lasting more than 30 minutes/time, and the intensity of each exercise is moderate or above)
9.	Often smoking	0= Yes, 1= No (Smoking 5 cigarettes/day, 20 days/month, and continue for more than 3 months)
10.	Alcoholism	0= Yes, 1= No (Drink alcohol for 100g/day, 20 days/month, and last for more than 3 months)
11.	Excessive smartphone use	0= Yes, 1= No (Use your mobile phone to brush short videos or play games for ≥4h/day (excluding normal use of mobile phone communication), and last for more than 3 months).This duration-based criterion was employed as a practical indicator of excessive use in our study
12.	Father’s education	0= junior high school or below; 1= senior high school or technical secondary school, 2= university or above
13.	Maternal education	0= junior high school or below; 1= senior high school or technical secondary school, 2= university or above
14.	Household income	0= Rich (Annual income of $500,000), 1= General (100,000-500,000), 2= difference (<100,000)
15.	Household structure	0= core family, 1= extended family, 2= single parent, 3= reorganized family
16.	Siblings	0= non-only child, 1= only child
17.	Familial genetic history of psychosis	0= none, and 1= have

### Procedures

Before the survey, an extensive literature review, expert advice, and project design were considered to determine the research subjects, tools, and content. To ensure the smooth progress of the survey, a detailed training plan was developed for the surveyors prior to the investigation. The training covered the project background, survey locations, research subjects, tools, and key considerations. Surveyors were provided with standardized instructions for administering the questionnaires, along with explicit guidelines regarding time constraints. Data collection was carried out through group testing via the online platform.

### Ethical considerations

The study protocol was approved by the medical ethics committee of the Second Affiliated Hospital of Wannan Medical College (Number: WYEFYLS2023086). Ethical issues (Including plagiarism, informed consent, misconduct, data fabrication and/or falsification, double publication and/or submission, redundancy, etc.) have been completely observed by the authors.

### Data analysis

Data entry and analysis were performed using Epidata 3.2 and SPSS 21.0 software. Normally distributed data are presented as mean ± standard deviation (X ± s), and categorical data are presented as frequency and percentage (n, %). The χ² test was used for univariate analysis to identify factors influencing different depressive symptoms. One-way analysis of variance (ANOVA) was chosen to assess the differences between groups with different depressive symptoms, as it can compare mean differences among multiple groups. Additionally, multivariate logistic regression analysis was employed to examine the relationship between the basic characteristics of college students and depressive symptoms, as it can handle binary or multi-category dependent variables and adjust for potential confounding variables. Statistical significance was set at p < 0.05.

## Results

### Prevalence and correlates of depressive symptoms among college students: a CES-D scale analysis

The findings revealed a mean CES-D score of 13.44 ± 9.21. Among the participants, 875 exhibited depressive symptoms, resulting in a depression detection rate of 25.60%, while 340 individuals met the criteria for subthreshold depression, representing 9.97%. Examination of CES-D scores across various characteristics showed that males scored significantly higher than females (15.32 ± 9.86 vs. 12.21 ± 8.54, p-value < 0.01), and significant grade-related differences were observed, with senior students exhibiting the highest scores (16.21 ± 10.24) and freshmen the lowest (12.55 ± 9.00, p < 0.01) (**Table 2**). Elevated scores were strongly associated with frequent insomnia, drinking, smoking, excessive mobile phone gaming, or low family income (p-value < 0.01). Conversely, regular engagement in sports and satisfaction with one’s academic major were linked to substantially lower scores (p-value < 0.01). Additionally, students from extended and core families exhibited relatively lower scores (p-value < 0.05), while those with a family history of psychiatric disorders had higher scores (p-value < 0.01).

**Table 2:** Demographic analysis of depressive symptom scores (CES-D).

Variable	Total (*n* = 3,418)	CES-D Score (% ± SD)	t/z/χ^2^ value	(*p*-value)
Sex, *n* (%)				
Male,*n* (%)	1353 (39.58%)	15.32 ± 9.86	9.798	•••
Females, *n* (%)	2065 (60.42%)	12.21 ± 8.54		
specialty(%)				
Medicine	1567	13.24 ± 9.13	1.130	0.258
Non-medicine	1851	13.60 ± 9.28		
Student origin, *n* (%)				
Rural area	1652	13.60 ± 9.12	1.012	0.311
Town	1766	13.28 ± 9.29		
Seniority, *n* (%)				
Freshman	1461	12.55 ± 9.00	10.229	•••
Sophomore	1214	13.97 ± 9.32		
Junior	626	13.97 ± 9.08		
Senior	117	16.21 ± 10.24		
Profession satisfaction, *n* (%)				
Satisfied	1945	11.95 ± 8.90	66.128	•••
Normal	1361	15.18 ± 9.17		
Dissatisfactory	112	18.04 ± 9.86		
Insomnia,*n* (%)				
Not have	1886	11.42 ± 8.75	161.375	•••
Once in a while	1371	15.15 ± 8.73		
Often	161	22.53 ± 10.00		
Activeness, *n* (%)				
Yes	2154	12.82 ± 9.22	26.139	•••
Deny	1264	14.49 ± 9.11		
Frequent smoking, *n* (%)				
Yes	107	20.84 ± 10.66	72.838	•••
Deny	3311	13.20 ± 9.06		
Alcoholism, *n* (%)				
Yes	95	20.89 ± 10.04	65.230	•••
Deny	3323	13.22 ± 9.10		
Excessive smartphone use, *n* (%)				
Yes	2368	14.04 ± 9.18	33.576	•••
Deny	1050	12.07 ± 9.15		
Father’s literacy level, *n* (%)				
Junior school (7^th^-9^th^ grade) and below	2037	13.28 ± 9.10	0.762	0.467
High school or technical secondary school (10^th^-12^th^	832	13.65 ± 9.14		
University and above	549	13.70 ± 9.73		
Maternal education level, n (%)				
Junior school (7^th^-9^th^ grade) and below	2386	13.30 ± 9.05	1.956	0.142
High school or technical secondary school (10^th^-12^th^	655	14.07 ± 9.47		
University and above	377	13.18 ± 9.74		
Household income, n (%)				
Prosperous	425	13.02 ± 9.78	35.980	●●●
Middle class	2305	12.73 ± 8.79		
Low	688	16.07 ± 9.76		
Family type, n (%)				
Core (normal) family	2438	13.47 ± 9.26	3.262	●
Expanded family	729	12.87 ± 8.93		
Single-parent family	176	14.36 ± 8.91		
Reconstituted family	75	15.87 ± 10.43		
Siblings ranking, n (%)				
The only child	1097	13.67 ± 9.46	1.001	0.317
With siblings	2321	13.33 ± 9.10		
Family genetic history of psychiatric disorders				
yes	299	15.64 ± 9.63	18.857	•••
deny	3119	13.23 ± 9.14		

*p-*value ≤ 0.05 (•), ≤0.01(••), ≤0.001 (•••).

### Characteristics analysis of college students with different depressive symptoms

The incidence of subthreshold depression and clinical depression across different demographic and behavioral groups is presented in **Table 3**. One-way ANOVA revealed significant disparities in the distribution of depressive symptoms among the groups.

**Table 3:** Univariate analysis of college students with different depressive symptoms.

Influencing factor		*n*	Without depression (%)	Subthreshold depression (%)	Depressed *n* (%)	χ^2^value	(*p*-value)
Sex	man	1353	767(56.69)	121(8.94)	465(34.37)	90.474	0.000
	woman	2065	1436(69.54)	219(10.61)	410(19.85)		
Origin of the student	rural area	1652	1062(64.29)	168(10.17)	422(25.54)	0.176	0.916
	town	1766	1141(64.61)	172(9.74)	453(25.65)		
Seniority	freshman	1461	1004(68.72)	154(10.54)	303(20.74)	40.686	0.000
	sophomore	1214	746(61.45)	112(9.23)	356(29.32)		
	junior	626	389(62.14)	67(10.70)	170(27.16)		
	senior	117	64(54.70)	7(5.98)	46(39.32)		
Profession satisfaction	satisfied	1945	1383(71.10)	154(7.92)	408(20.98)	94.133	0.000
	same as	1361	770(56.58)	170(12.49)	421(30.93)		
	discontent	112	50(44.64)	16(14.29)	46(41.07)		
Insomnia	not have	1886	1388(73.60)	143(7.58)	355(18.82)	232.991	0.000
	Once in a while	1371	773(56.38)	179(13.06)	419(30.56)		
	often	161	42(26.09)	18(11.18)	101(62.73)		
Activeness	yes	2154	1449(67.27)	183(8.50)	522(24.23)	23.754	0.000
	deny	1264	754(59.65)	157(12.42)	353(27.93)		
Smoking	yes	107	41(38.32)	7(6.54)	59(55.14)	50.646	0.000
	deny	3311	2162(65.30)	333(10.06)	816(24.64)		
Alcoholism	yes	95	31(32.63)	13(13.69)	51(53.68)	46.830	0.000
	deny	3323	2172(65.36)	327(9.84)	824(24.80)		
Excessive smartphone use	yes	2368	1463(61.78)	270(11.40)	635(26.82)	29.383	0.000
	deny	1050	740(70.47)	70(6.67)	240(22.86)		
Household income	prosperous	425	283(66.59)	34(8.00)	108(25.41)	64.420	0.000
	Middle class	2305	1565(67.90)	217(9.41)	523(22.69)		
	low	688	355(51.60)	89(12.94)	244(35.46)		
Family type	Core (normal) family	2438	1575(64.60)	223(9.15)	640(26.25)	19.259	0.004
	Expanded family	729	489(67.08)	83(11.38)	157(21.54)		
	Single parent family	176	99(56.25)	25(14.20)	52(29.55)		
	Reconstituted family	75	40(53.33)	9(12.00)	26(34.67)		
Siblings	yes	1097	696(63.45)	99(9.02)	302(27.53)	3.987	0.136
	deny	2321	1507(64.93)	241(10.38)	573(24.69)		
History of psychiatric disorders	yes	299	158(52.84)	29(9.70)	112(37.46)	24.872	0.000
	deny	3119	2045(65.57)	311(9.97)	763(24.46)		

*p-*value ≤ 0.05 (•), ≤0.01(••), ≤0.001 (•••).

Demographic factors: Significant differences were observed by gender (χ² = 90.474, p < 0.001) and academic seniority (χ² = 40.686, p < 0.001). Females exhibited a higher incidence of subthreshold depression (10.61% vs. 8.94%), while males had a substantially higher incidence of clinical depression (34.37% vs. 19.85%). The incidence of clinical depression was highest among senior students (39.32%), followed by sophomores (29.32%) and juniors (27.16%), with freshmen showing the lowest rate (20.74%).

Academic and family factors: Major satisfaction was a significant factor (χ² = 94.133, p < 0.001). Students dissatisfied with their major had the highest incidence of both subthreshold (14.29%) and clinical depression (41.07%). Family income (χ² = 64.420, p < 0.001) and a history of psychiatric disorders (χ² = 24.872, p < 0.001) were also significant predictors. Students from low-income families and those with a family history of mental illness showed elevated rates of clinical depression (35.46% and 37.46%, respectively).

Behavioral factors: Several behavioral factors were significantly associated with depressive symptoms. These included insomnia (χ² = 232.991, p < 0.001), lack of regular exercise (χ² = 23.754, p < 0.001), frequent smoking (χ² = 50.646, p < 0.001), frequent alcohol consumption (χ² = 46.830, p < 0.001), and excessive smartphone use (χ² = 29.383, p < 0.001). Students with excessive smartphone use had a higher incidence of both subthreshold (11.40% vs. 6.67%) and clinical depression (26.82% vs. 22.86%).

### Analysis of influencing factors on different depression symptoms in college students

Conducting an unordered multivariate logistic regression analysis, we used various depression symptoms in college students as dependent variables.The model included the following eleven independent variables: gender, academic seniority, major satisfaction, insomnia, regular physical exercise, frequent smoking, frequent alcohol consumption, excessive smartphone use, household income, family structure, and family history of psychiatric disorders. The “no depression group” served as the baseline for comparison, helping to elucidate the impact of distinct group characteristics on different depression symptoms (**Table 4**).

**Table 4:** Logistic Multivariate analysis of the factors influencing different depressive symptoms among college students.

Argument (reference)		Subthreshold depression			Depressed		
		*B*	*p-*value	*OR(95%CI)*	*B*	*p-*value	*OR(95%CI)*
Gender (female)	man	0.204	0.122	1.226(0.947-1.587)	0.846	•••	2.331(1.945-2.793)
Seniority (Final year)	freshman	0.351	0.400	1.420(0.627-3.216)	-0.847	•••	0.429(0.276-0.665)
	sophomore	0.255	0.542	1.290(0.569-2.927)	-0.466	•	0.628(0.407-0.969)
	junior	0.397	0.352	1.487(0.644-3.433)	-0.616	•••	0.540(0.342-0.851)
Profession satisfaction (dissatisfactory)	satisfied	-0.756	•	0.470(0.255-0.864)	-0.915	•••	0.401(0.253-0.635)
	Normal	-0.302	0.326	0.739(0.405-1.351)	-0.390	0.095	0.677(0.429-1.070)
Insomnia (often)	None	-1.160	•••	0.314(0.173-0.567)	-1.959	•••	0.141(0.094-0.211)
	Once in a while	-0.464	0.120	0.629(0.350-1.129)	-1.298	•••	0.273(0.183-0.407)
Activeness (no)	yes	-0.337	•••	0.714(0.556-0.917)	-0.339	•••	0.713(0.591-0.859)
Smoking(no)	yes	-0.398	0.422	0.672(0.255-1.772)	0.625	•	1.869(1.094-3.194)
Alcohol drinking(no)	yes	1.367	•••	3.925(1.769-8.711)	0.935	••	2.548(1.419-4.576)
Excessive smartphone use (no)	yes	0.499	•••	1.647(1.237-2.193)	0.160	0.099	1.173(0.971-1.419)
Household income (poor)	Prosperous	-0.571	•	0.565(0.363-0.878)	-0.470	••	0.625(0.461-0.847)
	Normal	-0.500	•••	0.607(0.457-0.805)	-0.566	•••	0.568(0.461-0.700)
Family type (reorganized family)	Core (normal) family	-0.268	0.488	0.765(0.359-1.630)	-0.271	0.332	0.763(0.441-1.319)
	Expanded family	-0.143	0.718	0.866(0.398-1.889)	-0.494	0.090	0.610(0.345-1.080)
	Single-parent family	0.169	0.702	1.184(0.498-2.818)	-0.163	0.623	0.850(0.444-1.626)
History of psychiatric disorders	none	-0.156	0.470	0.855(0.560-1.307)	-0.499	•••	0.607(0.460-0.802)

*p-*value ≤ 0.05 (•), ≤0.01(••), ≤0.001 (•••).

As indicated in **Table 4**, using the “no depression group” as a baseline, major satisfaction, insomnia, regular exercise, frequent drinking, and family income emerged as significant and consistent predictors for both subthreshold depression and clinical depression. Conversely, gender, academic seniority, frequent smoking, and a family history of mental illness demonstrated a notable and specific impact, significantly increasing the risk for clinical depression but not for subthreshold depression. Furthermore, excessive smartphone use displayed a distinct pattern, being a significant predictor specifically for subthreshold depression but not for clinical depression.

Specifically, males exhibited a higher susceptibility to depressive symptoms. Students dissatisfied with their majors, those experiencing frequent insomnia and alcohol consumption, and individuals with lower family income were more prone to both subthreshold depression and depression. The risk of subthreshold depression was 3.925 times higher for frequent drinkers (defined as consuming >100 g of alcohol per day, for ≥20 days/month, over >3 months) compared to social drinkers (the reference group, which included non-drinkers and those who drank less frequently or in lower quantities), and the risk of depression was 2.548 times higher. Additionally, college students with excessive smartphone use had a 1.647 times higher risk of experiencing subthreshold depression.

## Discussion

College students, in the transition from adolescence to early adulthood, undergo significant changes in their academic and lifestyle routines. This transition makes them particularly vulnerable to external influences, which can contribute to the onset of negative emotions, such as depression, making them a high-risk demographic. Our study aimed to understand the prevalence of depression, subthreshold depression, and the absence of depression among college students. The findings revealed that 9.97% of participants experienced subthreshold depression, while 25.60% experienced depression. Interestingly, there was considerable diversity within the group, supporting the growing perspective in research that views depression as a spectrum disorder. This study was designed to address the following research questions: (1) What are the prevalence rates of depression and subthreshold depression among Chinese university students? (2) How do demographic factors (e.g., gender, academic year, family background) and behavioral patterns (e.g., exercise, smartphone use, substance use) differentially influence the risk of subthreshold depression versus clinical depression? (3) How do these observed associations compare with and contribute to the existing literature on depressive symptoms in young adults?

The detection rate of subthreshold depression in this investigation was lower than that found in a survey across 11 European countries (22.9%) (15), but similar to the 9.30% rate reported in a study by Zhang et al. in China (16). On the other hand, the detection rate of depression was higher than that reported by Chang et al., among college students in southwestern China (20.9%) (17). These variations could be attributed to the absence of a standardized definition for subthreshold depression, leading researchers to use different criteria based on their specific study objectives and assessment methodologies. This lack of consensus, as highlighted in systematic reviews (7, 8), underscores the challenge of direct cross-study comparisons and the need for more standardized criteria in future research.

In our current study, we defined subthreshold depression among college students based on their emotional symptoms and CES-D scores falling within the range of 16 to 19. A similar approach was employed by Yin et al., who defined subthreshold depression with CES-D scores ranging from 24 to just under 29 (11). The use of different cutoff scores for subthreshold depression across studies is common and reflects the current lack of a standardized definition for this condition. Crucially, a cutoff score of ≥16 on the CES-D is a widely used and validated benchmark in epidemiological research to identify individuals with significant depressive symptomatology. For instance, this cutoff has been employed to screen for potential clinical depression in smoking cessation trials (18), to investigate the association between dietary tryptophan and depression in large cohorts of women (19), and as a primary risk factor in studies of COVID-19 breakthrough infections (20). Therefore, while the specific criteria may vary (as seen in the comparison withYin et al.), our use of the CES-D score range of 16–19 to define subthreshold depression is grounded in established research practices and allows for the identification of a student population experiencing substantial depressive symptoms worthy of clinical attention. This variability in the literature underscores the necessity for additional research to establish more harmonized assessment criteria for subthreshold depression.

The current study highlights notable distinctions among college students exhibiting different depression symptoms in relation to gender, academic year, professional satisfaction, and family background. Students from families characterized by lower economic income, single-parent households, and blended families demonstrated relatively higher rates of both subthreshold depression and full-blown depression. This aligns with existing literature suggesting that socioeconomic stressors and less stable family environments are significant risk factors for mental health issues in young adults (17). The prevalence of depression was found to be higher in males compared to females, indicating that males face a 2.331 times greater risk of experiencing depression. However, it is essential to acknowledge that significant discrepancies exist in research findings regarding gender differences in depression. For instance, Marcelo and colleagues proposed that females are more susceptible to subthreshold depression and experience more pronounced depression symptoms and sleep problems (21). Our finding of higher clinical depression in males resonates with the study by Cavanagh et al. (22), which may be explained by differing help-seeking behaviors, socialization patterns, or expression of depressive symptoms (e.g., irritability vs. sadness) across genders.

Sassarini et al. reported a higher prevalence of depression among females compared to males, as supported by their studies. Conversely, J. Oldham et al. found higher rates of depression in males, a result further supported by Cavanagh et al., where males reported more depression-related symptoms than females (22–24). These varying findings highlight the importance of addressing gender differences in depression symptoms, suggesting that gender roles may contribute to disparities in the understanding of depression. Moreover, our observations revealed a higher incidence of subthreshold depression in females compared to males. This discrepancy could be linked to greater emotional resilience among females and a tendency for males to exhibit biased self-assessment concerning social role expectations, which may render them more susceptible to significant negative emotions when confronted with adverse stimuli.

We acknowledge that the distribution of students across different academic majors (e.g., medical vs. non-medical) and the specific stresses associated with these majors could be one potential factor influencing the observed gender differences in our study. However, this study was not designed to disentangle the complex interplay between gender, academic discipline, and depression. Future research that delves into major-specific stressors and their interaction with gender would be valuable to clarify this issue.

The prevalence of depression was highest among senior students, and multivariate regression analysis confirmed that seniority was a significant independent predictor of depression, with students in their final year facing the highest risk. The detection rate of subthreshold depression is higher in lower-grade students compared to those in their graduating (internship) year, which contrasts with the findings of K.K. Jha et al. (25). During the COVID-19 pandemic, first- and second-year students were immediately affected by the outbreak, leading to a higher subthreshold depression rate among juniors. We believe that seniors face increased self-imposed demands, such as the pressure of graduation, employment, or further studies, and more apparent changes in their emotional lives. Overall, seniors experience higher stress levels than juniors. This also suggests that more attention and intervention should be directed toward students in their graduating (internship) year to address emerging psychological issues.

Students who are satisfied with their chosen profession have lower rates of depression and subthreshold depression. Multiple logistic regression analysis indicates that major satisfaction significantly predicts both subthreshold depression and depression. Sokol et al. considered low professional identification an important cause of depression (26). We believe that students’ satisfaction with their chosen profession can stimulate positive learning emotions, foster professional identity, and increase self-efficacy. Self-efficacy involves students’ evaluation of their own abilities, and when students’ self-evaluations are positive, their risk of depression decreases. This is consistent with the concept of ‘temporal self-appraisal’ proposed by Sokol & Serper, where a negative view of one’s self-continuity is linked to depression (26). Therefore, strengthening education on professional identity for students, exploring and integrating the fusion of individual identity with the nature of the profession, and enhancing the fit between students and their majors, is not only necessary for professional education but also an important measure to promote students’ psychological well-being.

The daily behavioral patterns of college students significantly impact various depression symptoms. College students who frequently experience insomnia, smoke, consume alcohol, and excessively use smartphones show significant differences across groups in terms of no depression, subthreshold depression, and depression. Students who engage in regular physical exercise tend to have lower rates of subthreshold depression and depression. Logistic multiple-factor analysis reveals that insomnia, regular physical exercise, and frequent alcohol consumption are significant predictors of both subthreshold depression and depression. Excessive smartphone use significantly predicts subthreshold depression, while frequent smoking significantly predicts depression.

Insomnia is considered a concomitant symptom of depression, and good sleep is a positive factor in preventing subthreshold depression and depression. This study found that frequent smoking (more than 5 cigarettes per day, ≥20 days per month, lasting for more than 3 months) and frequent alcohol consumption (more than 100g per day, ≥20 days per month, lasting for more than 3 months) are risk factors for both depression and subthreshold depression. Research has shown a strong correlation between unhealthy behaviors, such as smoking, drinking, and depressive emotions (27). College students’ psychological states are still maturing, and when faced with situational stress, they may resort to inappropriate coping mechanisms and forms of expression, leading to impulsive behaviors like smoking and drinking. These behaviors may not effectively alleviate negative emotions. Instead, they may form a vicious cycle where negative emotions promote substance use, which in turn exacerbates depression, as suggested by studies on the co-morbidity of depression and substance use (27). Conversely, regular physical exercise (sports for ≥3 days per week, ≥30 minutes each session, at moderate intensity) is a protective factor against depression and subthreshold depression. Physical exercise promotes adolescents’ emotional regulation and non-cognitive abilities, such as psychological resilience (28). However, some studies suggest an inverse relationship between exercise intensity and psychological reactions, with high-intensity exercise possibly affecting mental health (29). Our study confirms the benefit of moderate exercise, supporting the findings of Paolucci et al. that exercise reduces depression but intensity matters (29). Therefore, further analysis and research are needed to understand the impact of exercise intensity and methods on depression.

Compared to normal smartphone users, students who engage in excessive smartphone use (operationalized in our study as usage for watching short videos or playing games for ≥4 hours per day over more than 3 months) had a 1.647 times higher risk of developing subthreshold depression. The widespread phenomenon of smartphone usage among students, especially excessive use for watching videos or playing games, may impact their mental health. This is likely due to the complexity of information on the internet, where it can be difficult to distinguish between true and false information, and some content may be negatively misleading. Most smartphone usage for browsing online content lacks a clear purpose, often resulting in passive information consumption. On the other hand, excessive smartphone use can lead students to become immersed in gaming, reducing cognitive abilities, decreasing emotional stability, and making them more susceptible to negative emotions. Game addiction is closely related to depression, anxiety, and negative coping styles, which can further increase gaming behavior, impair social functioning, and hinder recovery (30, 31). Our finding that excessive smartphone use specifically predicts subthreshold depression is noteworthy. It suggests that prolonged immersion in passive content consumption or gaming may act as a precursor to depressive states, potentially through mechanisms like social displacement, reduced real-world reinforcement, and sleep disruption (30, 32).

In previous studies, more attention has been given to individual differences between healthy individuals and those with depression. This study adopts a spectrum perspective on depression, focusing on the occurrence of no depression, subthreshold depression, and depression among college students. The analysis explores the impact of daily behavioral patterns on various depression symptoms. The findings indicate that students dissatisfied with their selected profession, those in their graduating year, and male students have a higher risk of experiencing depression. Smoking more than 5 cigarettes per day, consuming alcohol in amounts greater than 100g per day for ≥20 days per month, and using smartphones to watch short videos or play games for ≥4 hours per day are associated with a higher likelihood of developing depressive symptoms (32). Additionally, engaging in physical exercise for ≥3 days per week, for ≥30 minutes each time, at a moderate intensity, can reduce the risk of subthreshold depression and depression.

This study does have some limitations. First, the sample only comes from a group of university students in a specific region, which may not be representative of the nationwide prevalence of depression symptoms among university students. Second, the study relies on self-report questionnaires (such as the CES-D scale) to assess depressive symptoms. Although these scales are widely used, self-report data may be influenced by participants’ social desirability bias or misinterpretations of symptoms, which could lead to data bias. Third, the measurement of excessive smartphone use was based solely on self-reported screen time (≥4 hours per day). While this provides a practical and quantifiable measure, it does not capture the core psychological components of behavioral addiction (e.g., loss of control, tolerance, and withdrawal) and should be interpreted as a proxy for excessive use rather than a clinical diagnosis of addiction. Fourth, and relatedly, the CES-D scale measures symptoms over the past week and was not supplemented with a structured clinical interview. Therefore, our study could not ascertain whether the depressive symptoms met the duration (e.g., persisting for at least two weeks as required by DSM-5) and full symptom criteria for a major depressive episode. Consequently, the ‘depression’ and ‘subthreshold depression’ categories identified in this study reflect levels of self-reported symptomatology rather than clinical diagnoses. Fifth, the study did not employ diagnostic tools to rule out other psychiatric, neurological, or developmental conditions, which could co-occur with or influence depressive symptoms. Additionally, the cross-sectional nature of our design prevents us from drawing causal inferences about the relationships between risk factors and depression symptoms. Furthermore, the comparison groups (non-depressive, subthreshold depression, and depression) were not matched on baseline characteristics such as age, sex, and education. Although these variables were statistically controlled for in our multivariate logistic regression model, the lack of matching remains a consideration. While this study found that regular physical exercise is associated with a reduced risk of depressive symptoms, it did not delve into the specific effects of different intensities or types of exercise on mental health. Future research could further explore the preventive effects of various types of physical activities (such as aerobic exercise, strength training, etc.), as well as their frequency and intensity, on depressive symptoms among university students. Longitudinal studies are needed to establish causality and to track the potential progression from subthreshold to clinical depression.

## Conclusion

In conclusion, this study provides clear evidence that depression among Chinese university students exists across a spectrum, with a high prevalence of both clinical (25.60%) and subthreshold depression (9.97%). Our findings identify distinct risk profiles for these conditions. Key demographic predictors of clinical depression include male gender, senior academic standing, and lower family income. Crucially, we identified modifiable behavioral factors: regular moderate-intensity exercise and major satisfaction serve as significant protective factors. Conversely, frequent insomnia, smoking, and alcohol consumption are substantial risk factors. A novel finding of this study is that excessive smartphone use specifically predisposes students to subthreshold depression, positioning it as a potential precursor to more severe clinical states. These results underscore the necessity of comprehensive and tailored interventions that address both demographic vulnerabilities and daily lifestyle choices to mitigate the burden of depression in the university student population.

## Data Availability

The raw data supporting the conclusions of this article will be made available by the authors, without undue reservation.

## References

[1] APA, Diagnostic and statistical manual of mental disorders: DSM-5™. In: Diagnostic and statistical manual of mental disorders: DSM-5™, 5th ed. Arlington, VA, US: American Psychiatric Publishing, Inc.. p. xliv, 947–xliv, 947.

[2] Vos T Lim SS Abbafati C Abbas KM Abbasi M Abbasifard M . Global burden of 369 diseases and injuries in 204 countries and territories, 1990&x2013;2019: a systematic analysis for the Global Burden of Disease Study 2019. Lancet. (2020) 396:1204–22. doi: 10.1016/S0140-6736(20)30925-9, PMID: 33069326 PMC7567026

[3] Yueqin H Yu W Hong W Zhaorui L Xin Y Jie Y . Prevalence of mental disorders in China: a cross-sectional epidemiological study. Lancet Psychiatry. (2019) 6:211–24. doi: 10.1016/S2215-0366(18)30511-X, PMID: 30792114

[4] Dan-Dan X Wen-Wang R Xiao-Lan C Si-Ying W Feng R Weng-Ian C . Prevalence of depressive symptoms in primary school students in China: A systematic review and meta-analysis. J Affect Disord. (2020) 268:20–7. doi: 10.1016/j.jad.2020.02.034, PMID: 32158003

[5] Mi ZH Guangsheng ZH Scott R Kaleigh K Hao X . Depressive symptoms of chinese children: prevalence and correlated factors among subgroups. Int J Environ Res Public Health. (2018) 15:283. doi: 10.3390/ijerph15020283, PMID: 29414881 PMC5858352

[6] Aldao A Nolen-Hoeksema S Schweizer S . Emotion-regulation strategies across psychopathology: A meta-analytic review. Clin Psychol Rev. (2010) 30:217–37. doi: 10.1016/j.cpr.2009.11.004, PMID: 20015584

[7] Rodríguez MR Nuevo R Chatterji S Ayuso-Mateos JL . Definitions and factors associated with subthreshold depressive conditions: a systematic review. BMC Psychiatry. (2012) 12:181. doi: 10.1186/1471-244X-12-181, PMID: 23110575 PMC3539957

[8] Wesselhoeft R Sørensen MJ Heiervang ER Bilenberg N . Subthreshold depression in children and adolescents - a systematic review. J Affect Disord. (2013) 151:7–22. doi: 10.1016/j.jad.2013.06.010, PMID: 23856281

[9] Cuijpers P Graaf RD Dorsselaer SV . Minor depression: risk profiles, functional disability, health care use and risk of developing major depression. J Affect Disord. (2004) 79:71–9. doi: 10.1016/S0165-0327(02)00348-8, PMID: 15023482

[10] Bertha EA Bala´zs J . Subthreshold depression in adolescence: a systematic review. Eur Child Adolesc Psychiatry. (2013) 22:589–603. doi: 10.1007/s00787-013-0411-0, PMID: 23579389

[11] Xiaoke Y Zhiwei T Ali A . The effect of mixed teaching mode based on network open course and intelligent teaching platform on alleviating students’ psychological anxiety. Int J Neuropsychopharmacol. (2022) 25:A76–7. doi: 10.1093/ijnp/pyac032.104

[12] Berk M . Biomarkers in psychiatric disorders: status quo, impediments and facilitators. World Psychiatry. (2023) 22:174–6. doi: 10.1002/wps.21071, PMID: 37159364 PMC10168162

[13] Hankin BL Young JF Abela JRZ Smolen A Jenness JL Gulley LD . Depression from childhood into late adolescence: Influence of gender, development, genetic susceptibility, and peer stress. J Abnorm Psychol. (2015) 124:803–16. doi: 10.1037/abn0000089, PMID: 26595469 PMC4662048

[14] Zimmermann P Iwanski A . Emotion regulation from early adolescence to emerging adulthood and middle adulthood: Age differences, gender differences, and emotion-specific developmental variations. Int J Behav Dev. (2014) 38:182–94. doi: 10.1177/0165025413515405

[15] Balázs J Mónika M Keresztény A Hoven CW Carli V Wasserman C . Adolescent subthreshold-depression and anxiety: psychopathology, functional impairment and increased suicide risk. J Child Psychol Psychiatry. (2013) 54:670–7. doi: 10.1111/jcpp.12016, PMID: 23330982

[16] Shaohua Z Biao S Ying L Tingting P . Emotion Regulation Strategies in Adolescents with Different Depressive Symptoms. J Psychol Sci.. (2020) 43:1296–303. doi: 10.16719/j.cnki.1671-6981.20200603

[17] Hong C Ying W Si-qi L Yun Z Qing-ping X Xiong-fei P . Prevalence of depressive and anxiety symptoms among medical students, southwest China. Modern Prev Med. (2015) 42:3544–7. doi: CNKI:SUN:XDYF.0.2015-19-029

[18] Tidey JW Pacek L Koopmeiners JS Vandery R Donny E . Effects of 6-week use of reduced-nicotine content cigarettes in smokers with and without elevated depressive symptoms. Nicotine Tobacco Res. (2017) 19:59–67. doi: 10.1093/ntr/ntw199, PMID: 27613885 PMC5157715

[19] Hitomi S Asakura K Kobayashi S Nojima M Satoshi S . Association between habitual tryptophan intake and depressive symptoms in young and middle-aged women. J Affect Disord. (2018) 231:44–50. doi: 10.1016/j.jad.2018.01.029, PMID: 29438897

[20] Shimada Y Hori S Fukuda H Katsuta N Saita M Ohno M . A matched case-control study on the attributable risk of CES-D positivity to the incidence of COVID-19 breakthrough infections. Environ Occup Health Pract. (2024) 6. doi: 10.1539/eohp.2024-0007-OA, PMID: 40135244 PMC11933622

[21] Crockett MA Martínez V Jiménez-Molina A . Subthreshold depression in adolescence: Gender differences in prevalence, clinical features, and associated factors. J Affect Disord. (2020) 272:269–76. doi: 10.1016/j.jad.2020.03.111, PMID: 32553367

[22] Cavanagh A Wilson CJ Kavanagh DJ Caputi P . Analysis of depression status and its risk factors among college freshmen. China Public Health. (2017) 33:29–38. doi: 10.1097/HRP.0000000000000128, PMID: 28059934

[23] Sassarini DJ . Depression in midlife women. Maturitas. (2016) 94:149–54. doi: 10.1016/j.maturitas.2016.09.004, PMID: 27823736

[24] Oldham J Wang F Berkstresser B Lanois C Meehan W Howell D . Male Sex Predicts Higher Depression Scores Among Healthy Collegiate Athletes. Neurology. (2019) 93:S10–S10. doi: 10.1212/01.wnl.0000580924.24962.c7

[25] Jha KK Singh SK Nirala SK Kumar C Kumar P Aggrawal N . Prevalence of Depression among School-going Adolescents in an Urban Area of Bihar, India. Indian J Psychol Med. (2017) 39:287–92. doi: 10.4103/0253-7176.207326, PMID: 28615762 PMC5461838

[26] Sokol Y Serper M . Temporal self appraisal and continuous identity: Associations with depression and hopelessness. J Affect Disord. (2017) 208:503–11. doi: 10.1016/j.jad.2016.10.033, PMID: 27832930

[27] Xiuzhen C Zhenrong J Xiaojuan Y . Effect of life events, self-esteem and depression on suicidal ideation in college students. Chi J Health Psy. (2020) 28(10):1557–61. doi: 10.13342/j.cnki.cjhp.2020.10.027

[28] Yan S Zhou H . Review and outlook of the three major research topics of Sports and personality. Sports Sci. (2017) 37(7):60–72. doi: 10.16469/j.css.201707008

[29] Paolucci EM Loukov D Bowdish DME Heisz JJ . Exercise reduces depression and inflammation but intensity matters. Biol Psychol. (2018) 133:79–84. doi: 10.1016/j.biopsycho.2018.01.015, PMID: 29408464

[30] Chen IH Lee ZH Dong XY Gamble JH Feng HW . The influence of parenting style and time management tendency on internet gaming disorder among adolescents. Int J Environ Res Public Health.. (2020) 17:9120. doi: 10.3390/ijerph17239120, PMID: 33291336 PMC7730530

[31] Sussman CJ Harper JM Stahl JL Weigle P . Internet and video game addictions: diagnosis, epidemiology, and neurobiology. Child Adolesc Psychiatr Clinics. (2018) 27:307–26. doi: 10.1016/j.chc.2017.11.015, PMID: 29502753

[32] Loton D Borkoles E Lubman D Polman R . Video game addiction, engagement and symptoms of stress, depression and anxiety: the mediating role of coping. Int J Ment Health Addict. (2016) 14:565–78. doi: 10.1007/s11469-015-9578-6

